# Evolutionary and functional analyses of *LRP5* in archaic and extant modern humans

**DOI:** 10.1186/s40246-024-00616-6

**Published:** 2024-05-27

**Authors:** Neus Roca-Ayats, Iago Maceda, Carlos David Bruque, Núria Martínez-Gil, Natàlia Garcia-Giralt, Mónica Cozar, Leonardo Mellibovsky, Wim Van Hul, Oscar Lao, Daniel Grinberg, Susanna Balcells

**Affiliations:** 1 Departament de Genètica, Microbiologia i Estadística and IBUB, Universitat de Barcelona, Barcelona, Spain; 2 Centro de Investigación Biomédica en Red de Enfermedades Raras (CIBERER) ISCIII, Barcelona, Spain; 3 Institut de Recerca Sant Joan de Déu (IRSJD), Barcelona, Spain; 4 CNAG, Centre Nacional d’Analisi Genòmic, C/ Baldiri I Reixach 4, 08028 Barcelona, Spain; 5 Barcelona Institute of Science and Technology (BIST), Barcelona, Spain; 6 Universitat Pompeu Fabra (UPF), Barcelona, Spain; 7 Unidad de Conocimiento Traslacional Hospitalaria Patagónica, Hospital de Alta Complejidad El Calafate - S.A.M.I.C., Santa Cruz, Argentina; 8 Musculoskeletal Research Group, IMIM (Hospital del Mar Medical Research Institute), Centro de Investigación Biomédica en Red en Fragilidad y Envejecimiento Saludable (CIBERFES), ISCIII, Departament de Genètica, Microbiologia i Estadística, UB, Barcelona, Spain; 9 Musculoskeletal Research Group, IMIM (Hospital del Mar Medical Research Institute), Centro de Investigación Biomédica en Red en Fragilidad y Envejecimiento Saludable (CIBERFES), ISCIII, Barcelona, Spain; 10 Center of Medical Genetics, University of Antwerp, 2650 Antwerp, Belgium; 11 Institute of Evolutionary Biology, CSIC-Universitat Pompeu Fabra, 08003 Barcelona, Spain

**Keywords:** *LRP5*, Bone mineral density, Neanderthal, Denisovan, Human evolution, Archaic introgression

## Abstract

**Background:**

The human lineage has undergone a postcranial skeleton gracilization (i.e. lower bone mass and strength relative to body size) compared to other primates and archaic populations such as the Neanderthals. This gracilization has been traditionally explained by differences in the mechanical load that our ancestors exercised. However, there is growing evidence that gracilization could also be genetically influenced.

**Results:**

We have analyzed the *LRP5* gene, which is known to be associated with high bone mineral density conditions, from an evolutionary and functional point of view. Taking advantage of the published genomes of archaic *Homo* populations, our results suggest that this gene has a complex evolutionary history both between archaic and living humans and within living human populations. In particular, we identified the presence of different selective pressures in archaics and extant modern humans, as well as evidence of positive selection in the African and South East Asian populations from the 1000 Genomes Project. Furthermore, we observed a very limited evidence of archaic introgression in this gene (only at three haplotypes of East Asian ancestry out of the 1000 Genomes), compatible with a general erasing of the fingerprint of archaic introgression due to functional differences in archaics compared to extant modern humans. In agreement with this hypothesis, we observed private mutations in the archaic genomes that we experimentally validated as putatively increasing bone mineral density. In particular, four of five archaic missense mutations affecting the first β-propeller of LRP5 displayed enhanced Wnt pathway activation, of which two also displayed reduced negative regulation.

**Conclusions:**

In summary, these data suggest a genetic component contributing to the understanding of skeletal differences between extant modern humans and archaic *Homo* populations.

**Supplementary Information:**

The online version contains supplementary material available at 10.1186/s40246-024-00616-6.

## Introduction

The average bone mass and strength for body size has decayed on the *Homo* lineage, leading to a gracilization of the human postcranial skeleton compared to other primates [1]. *Homo ergaster* is commonly considered to have given rise to the precursors of both the *Homo sapiens* and *Homo neanderthalensis* lineages [2], which diverged between 400 and 800 kya [3]. Neanderthals and Pleistocene modern humans generally exhibit greater robustness compared to Holocene humans, including living populations [4, 5].

Bone strength is a complex phenotype determined by bone mineral density (BMD), bone geometry, cortical thickness and porosity, trabecular bone morphology, and intrinsic properties of bony tissue [6, 7]. The reduction in postcranial skeletal strength is notably pronounced in the later Pleistocene or Holocene, and it has been observed in both the cortical structure of long bones and the micro-structure of trabecular bone (including thickness and bone volume fraction) [1, 8–10]. Skeletal fragility has been primarily explained by the lack of physical activity, as bones respond to physical activity demands by adding tissue and modifying cross-sectional distribution in the direction of highest bending strains (i.e., change in length per unit length) [8, 11–13]. Other factors, such as high fertility rates, have been related to the decrease in BMD in women regardless of their amount of physical activity [14].

Nevertheless, in addition to environmental and behavioral factors, BMD is shown to be a highly heritable complex trait in humans, with up to 80% of the variance explained by genetic factors [15]. So far, genome wide association studies (GWAS) have identified a large number of genomic regions statistically associated with BMD variability, overall explaining 20% of the total estimated genetic variance [16]. Rare monogenic forms of osteoporosis and high BMD provide another source for understanding the molecular pathways of BMD determination, highlighting genes and genetic variants with a significant impact on the BMD phenotype [17].

The high bone mass (HBM) phenotype in living humans is estimated by dual-energy X-ray absorptiometry (DXA). As DXA has not been feasible for assessing BMD in fossilized bones from the Middle Pleistocene era, the inquiry remains unresolved regarding whether these ancient populations exhibited high bone mineralization. Nevertheless, HBM estimated by peripheral quantitative computed tomography (pQCT) is characterized in extant modern humans (EMH) by substantially greater trabecular and cortical BMD, leading to a greater predicted cortical bone strength [18]. Analyses of actual bone volume through pQCT in human fossils from the Middle Pleistocene, specifically at the Sima de los Huesos site, considered Neanderthal ancestors [19, 20], have revealed substantially increased bone volume and skeletal weight (i.e. skeletal robusticity) [21, 22].

Low-density lipoprotein receptor-related protein 5 gene (*LRP5*), encoding the co-receptor of the Wnt/β-catenin pathway -a major bone anabolic pathway-, can be considered as one of the key genes regulating bone mass [23]. It was one of the first to show association with BMD in living humans [24] and is always one of the top hits in GWAS. Additionally, and notably, it bears rare variants producing extreme BMD phenotypes. The first *LRP5* mutation causing HBM (p.G171V) was described in 2002 [25]. Since then, several other heterozygous missense mutations have been described leading to the same phenotype. These are gain-of-function mutations, which result in a stimulation of osteoblastic bone formation [26]. All HBM-associated *LRP5* mutations identified are located in exons 2, 3 and 4, which collectively code for the first β-propeller domain of the protein, and reduce LRP5 binding affinity for the inhibitors sclerostin and DKK1 protein [27]. In contrast, *LRP5* mutations causing the osteoporosis pseudoglioma syndrome are scattered throughout the gene and are loss-of-function variants.

Whereas the genetic architecture of BMD in *Homo sapiens* is becoming unraveled, little is known in extinct populations (i.e. archaic species) such as Neanderthals, and the impact of introgressed BMD variants in EMH populations. It has been previously suggested that archaic introgression in allochthonous populations allows the introduction of genetic variants that have been positively selected in the archaic populations. Conversely, purifying selection could have more effectively acted against hybridization [28]. It has also been reported that in modern human ancient samples, archaic ancestry decreased over time, particularly in areas near genes, and this observation has been diversely interpreted as evidence of hybrid sterility or a consequence of differences in effective population sizes between modern humans and Neanderthals [28]. From a genetic point of view, it has been suggested that non-African populations are enriched for derived low BMD-associated alleles at SNPs ascertained from GWAS compared to sub-Saharan populations, and that population phenotypic heterogeneity is the result of differential selective pressures in non-African versus Sub-Saharan populations [29]. Therefore, if the *LRP5* gene plays a main role in the BMD phenotype, we would expect to observe a depletion of archaic introgression in the *LRP5* gene in non-African populations.

In light of the current debate on the evolutionary history of the BMD phenotype, in this work we studied the genetic variation of *LRP5* in EMH as well as in archaic populations that are currently genomically known in order to test the presence of signals of positive selection in EMH. We further analyzed the role of archaic introgression in the *LRP5* gene, as well as identified genetic variants present in archaic populations that putatively increase BMD, and further validated them both structurally and functionally.

## Material and methods

### Databases

#### 1000 genomes project

Polymorphism information of EMH was retrieved from the publicly available variant call format (VCF) files from 1000 Genomes Project phase 3 (http://ftp.1000genomes.ebi.ac.uk/vol1/ftp/release/20130502/; 1000G) [30]. This dataset was filtered to obtain polymorphic biallelic single nucleotide variants (SNVs) inside the region of the *LRP5* gene according to Gencode v39 ± 500 kb. The ancestral allele was extracted from the 1000G VCF files (AA flag in the INFO field). The Altai Neanderthal [31], Denisovan [32], Vindija Neanderthal [33] and Chagyrskaya 8 Neanderthal [34] genomes were downloaded in VCF format from the Max Planck Institute for Evolutionary Anthropology ftp site (http://cdna.eva.mpg.de/neandertal). We selected polymorphisms that fall inside the region previously described and performed the same data filtering as before. The final dataset was created by merging the five previous datasets and we dropped out all SNVs that were not genotyped in all present samples. All filterings and the final merging were performed using the bcftools program [35].

#### Base conservation

phyloP (“phylogenetic P-values”; http://compgen.cshl.edu/phast/) [36] conservation scores were retrieved from the Vertebrate Multiz Alignment & Conservation (46 Species) in UCSC (http://hgdownload.soe.ucsc.edu/goldenPath/hg19/phyloP46way/). PhyloP statistic quantifies the conservation of a position compared to that expected under neutral drift. Sites predicted to be conserved show positive scores, while fast-evolving sites have negative scores (see [36] for further details).

### Assessment of archaic introgressed regions in the *LRP5* locus

Two different modern human reference datasets were used to test for archaic introgression in the *LRP5* region. First, we used the Sprime scores calculated for the 1000G individuals available in Browning, S. [37], since it captures a wide diversity with respect to global geographically-distributed populations. We selected those SNVs present inside the *LRP5* gene boundaries. For every individual, we calculated the number of alleles predicted as introgressed by Sprime. We computed the number of differences between each haplotype from the 1000G project and the Altai Neanderthal and Denisovan genomes using the phased data from the VCF files from the 1000G [30]. Second, we analyzed the presence of archaic introgressed single nucleotide polymorphisms (SNPs) identified in the genomes from 27,566 Icelanders [38], to take advantage of a greater sample size within a single population.

### Evidences of positive selection in *LRP5* in EMH

Statistics of positive selection were retrieved from PopHumanScan [39]. We considered a classical statistic such as π for computing the average number of nucleotide differences per site [40]. We further considered tests covering different time scales. For tests identifying signatures of positive selection in the range of millions of years, we ascertained α, the proportion of substitutions that are adaptive [41]. For selection events taking place < 250 kya, we considered Fay and Wu’s H, the number of derived nucleotide variants at low and high frequencies compared with the number of variants at intermediate frequencies [42]. Finally, for events < 30 kya, we analyzed the integrated haplotype score (iHS), based on the frequency of alleles in regions of high linkage disequilibrium (LD) [43]. Each of them was computed at PopHumanScan on windows of 100 kb for each population of the 1000G, with the exception of iHS, which was computed using windows of 10 kb. PopHumanScan calculates an empirical p-value for the *LRP5* genomic region for each statistic and population. This is achieved by computing the frequency of genomic windows displaying a statistic value greater than the observed value. Therefore, the significance indicates that a *locus* is an outlier with respect to the rest of the genome.

We used BioMart [44] to retrieve autosomal genes of the human genome. In order to estimate a single value of each statistic for each gene and population, we computed the amount of shared fragments between each gene and the PopHumanScan database, and estimated a weighted mean. Standardization of each statistic for each population at the *LRP5* gene was conducted by constructing an empirical gene distribution estimated from a set of 1,098 autosomal genes with a similar length as *LRP5* (136.69 ± 20 kb).

### Statistical analyses

#### Scaling by majorizing a complicated function (SMACOF) analysis of the patterns of positive selection in 1000G at *LRP5* gene

A Euclidean distance matrix between pairs of populations from the 1000G was computed using the standardized values of each statistic of positive selection. The relationship between the different populations from the 1000G using the patterns of selection from the considered statistics was projected in two dimensions using ordinal SMACOF [45].

#### Weighted multidimensional scaling (wMDS) on EMH haplotypes and archaic genotypes at *LRP5*

In order to visualize the relationship between sequenced archaic individuals and haplotypes from individuals from the 1000G at the *LRP5* gene, we computed an identical by state (IBS) distance between pair of haplotypes (in the case of comparing two haplotypes from the 1000G), haplotype and scaled genotype (in the case of comparing 1000G individuals and archaic) and scaled genotypes (in the case of comparing two archaic individuals).

Given the unequal sample size of each continent and the archaic samples, in order to prevent biases in the estimated relationships by the MDS by sample size, we weighted the MDS using the function *wcmdscale* from the R package vegan [46], so the cluster of all EMH had the same weight as each archaic sample.

#### Analysis of the degree of conservation of derived alleles present either in the EMH lineage or the archaic lineage

We extracted the ancestral state of each SNP present at the *LRP5* gene from the 1000G VCF. In order to test whether SNPs in the EMH lineage occurred more often at evolutionary conserved genomic regions than SNPs that occurred in the archaic lineage, for each continent we sampled at random without replacement 1000 sets of four EMH individuals, matching the number of archaic samples. For each set we identified the polymorphic SNPs where the derived allele was present only in EMH (D_EMH) and the ones present only in archaic individuals (D_ARC). For each SNP we retrieved the PhyloP score and computed the difference between the mean amount of conservation in D_EMH with regards to D_ARC.

### Identification and selection of Neanderthal and Denisovan *LRP5* exonic variants

Neanderthal and Denisovan publicly available sequencing data (UCSC Genome Browser) were used to retrieve missense variants in *LRP5,* with a base quality score ≥ 23 and in a read with an alignment quality ≥ 150. Variants were filtered according to: (1) highly conserved positions; (2) damaging, according to Sorting intolerant from tolerant (SIFT) [47] and polymorphism phenotyping (Polyphen) [48]; 3) located in the HBM region of *LRP5* (i.e. first β-propeller). Finally, putatively functional variants (i.e., present in more than one Neanderthal individual, affecting the same protein residue, affecting a protein residue mutated in reported EMH HBM cases) were selected for further analyses. The presence and frequency of the variants in EMH were assessed using the gnomAD database.

### Model building and assessment

The sequence of the β-propeller region and the EGF-LIKE 1 domain of LRP5 from UniProt (O75197-1 (NP_002326.2)) was used to perform a sequence identity search in the Protein Data Bank (PDB) database. Five LRP6 templates were evaluated to make the model (PDB IDs: 3S94, 3SOB, 3SOQ, 3SOV, 4DG6 [49–51]; Supplementary Table S1). The X-Ray crystallography templates with a resolution of less than 2 Å (3SOB, 3SOQ, 3SOV) were selected and the molecular homology model (MHM) was generated using MODELLER version 9.22 [52]. The alignment of crystals and the LRP5 protein sequences was performed with the Modeller alignment program and hand-curated in the MEGA5 software [53] (Supplementary Fig. S1). In addition, the model was generated with a region of 7 residues of the DKK1 protein from the 3SOQ X-Ray crystallography. Model’s quality was assessed by discrete optimized potential energy (DOPE) [54], Qualitative Model Energy Analysis Distance Constraint (QMEANDisCo) [55], and Ramachandran plots [56]. Protein model is available in the Model Archive (10.5452/ma-1smp3). The UCSF Chimera program [57] was used for the structural visualization and interpretation of the variants.

### In silico mutagenesis and stability calculations

Protein variants were generated using FoldX 3.0 Beta 5.1 (foldx.crg.es) [58]. Repair PDB command was used to optimize the total energy of the protein to FoldX’s force field before residue changes were done. In silico mutagenesis was carried out using the BuildModel command, and each mutation was calculated five times. Protein interaction between LRP5 and DKK1 was calculated using the interaction command and protein stabilities, using the Stability command. ∆∆G values were estimated as the difference between the energy of the wild type protein and the average of five replicas for each protein variant. A threshold of 1.6 kcal/mol was considered, as it corresponds to twice the standard deviation calculated with FoldX.

### Cell culture

The Saos-2 cell line was used for luciferase reporter assays. It was obtained from the American Type Culture Collection (ATCC® htb-85™) and grown in Dulbecco’s Modified Eagle Medium (DMEM; Sigma-Aldrich), with 10% Fetal Bovine Serum (Gibco, Life Technologies) and 1% penicillin/streptomycin (Gibco, Life Technologies), at 37ºC and 5% of CO_2_.

### Plasmids and site-directed mutagenesis

The pGL3-OT luciferase reporter construct, the *Wnt1*-V5, *mesdc2, LRP5* and *DKK1*-FLAG expression vectors [59] were used. The *LRP5* mutations p.A67T, p.A67V, p.G171V (positive control), p.R186Q, p.M282R, and p.R291Q were introduced with the QuikChange Lightning Site-Directed Mutagenesis Kit (Agilent), following the manufacturer instructions. All the plasmids were validated by Sanger sequencing.

### In vitro luciferase reporter assay

Cells were seeded at a density of 1.5×10^5^ cells per well in 12-well plates. After 24 h, they were transfected with 1.072 µg of total DNA per well using the FuGENE HD reagent, according to manufacturer instructions (Promega): pGL3-OT (800 ng), pRL-TK (80 ng), containing the Renilla Luciferase gene, *Wnt1*-V5 (32 ng), *mesdc2* (64 ng), WT or mutated *LRP5* (64 ng) and, depending on the experiment, *DKK1*-FLAG (32 ng). When necessary, the empty pcDNA3 vector was used to adjust the total amount of DNA transfected. Forty-eight hours after transfection, cells were rinsed with PBS and lysed. The luciferase activity was measured using a Glomax Multi + luminometer (Promega), with the Dual-Luciferase® Reporter Assay System reagents (Promega). Each experiment was performed in triplicate and was repeated 3 times. Relative luciferase units (RLU, i.e., the ratio of the firefly luciferase activity over the Renilla luciferase activity) were calculated for each individual measurement and a one-way blocked ANOVA with Tukey HSD (honestly significant difference) multiple comparisons tests were performed using R software version 3.4.1 and *p*-values < 0.05 were considered significant. All the data was ascertained for normality, homoscedasticity and atypical data points.

## Results

### Evidence of differential selective pressures in *LRP5* within EMH

**Fig. 1:**
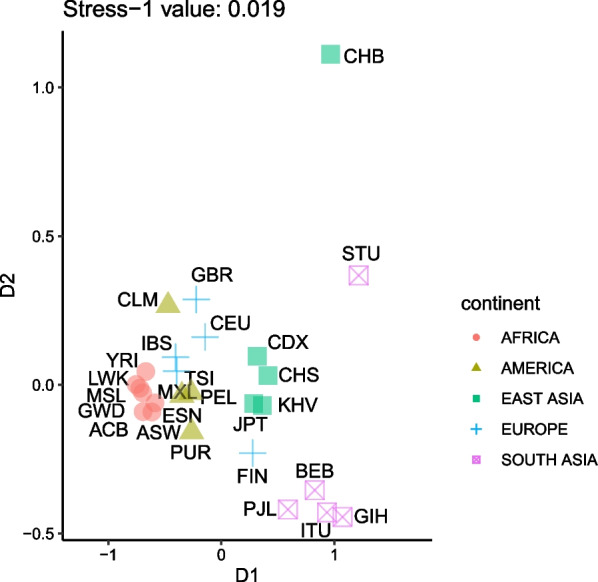
Relationship between the 1000G populations established by means of a non-metric ordinal SMACOF analysis using statistics of positive selection computed at the *LRP5* gene. YRI: Yoruba in Ibadan, Nigeria; LWK: Luhya in Webuye, Kenya; GWD: Gambian in Western Division; MSL: Mende in Sierra Leone; ESN: Esan in Nigeria; ACB: African Caribbean in Barbados; ASW: American's of African Ancestry in Southwest USA; CEU: Utah Residents (CEPH) with Northern and Western European ancestry; TSI: Toscani in Italia; FIN: Finnish in Finland; GBR: British in England and Scotland; IBS: Iberian population in Spain; CHB: Han Chinese in Beijing, China; JPT: Japanese in Tokyo, Japan; CHS: Southern Han Chinese; CDX: Chinese Dai in Xishuangbanna, China; KHV: Kinh in Ho Chi Minh City, Vietnam; GIH: Gujarati Indian from Houston, Texas; PJL: Punjabi from Lahore, Pakistan; BEB: Bengali from Bangladesh; STU: Sri Lankan Tamil from the UK; ITU: Indian Telugu from the UK; MXL: Mexican Ancestry from Los Angeles USA; PUR: Puerto Rican from Puerto Rica; CLM: Colombian from Medellín, Colombia; PEL: Peruvian from Lima, Peru

First, using non-metric ordinal SMACOF, we projected in two dimensions the relationships between the 1000G populations using ascertained statistics of positive selection computed at the *LRP5* gene (Fig. 1). 1000G populations tend to cluster according to their continental origin, particularly for the African (AFR) populations. The second dimension tends to distinguish CHB (Han Chinese from Beijing) and STU (Sri Lankan Tamil in the UK). Overall, the presence of geographic population substructure for summary statistics accounting for positive selection suggests that this gene could have been under different selective pressures among human populations.

Supporting this interpretation, popHumanScan reported evidence of genomic positive selection in Sub-Saharan populations for the α statistic (Supplementary Fig. S2A), accounting for the proportion of substitutions that are adaptive, and elevated values of iHS, summarizing (recent) departures in the allelic frequency given the observed haplotype length, in East Asian and, particularly, South Asian populations (Supplementary Fig. S2B).

### Identification of archaic introgression in EMH at the *LRP5locus*

**Fig. 2:**
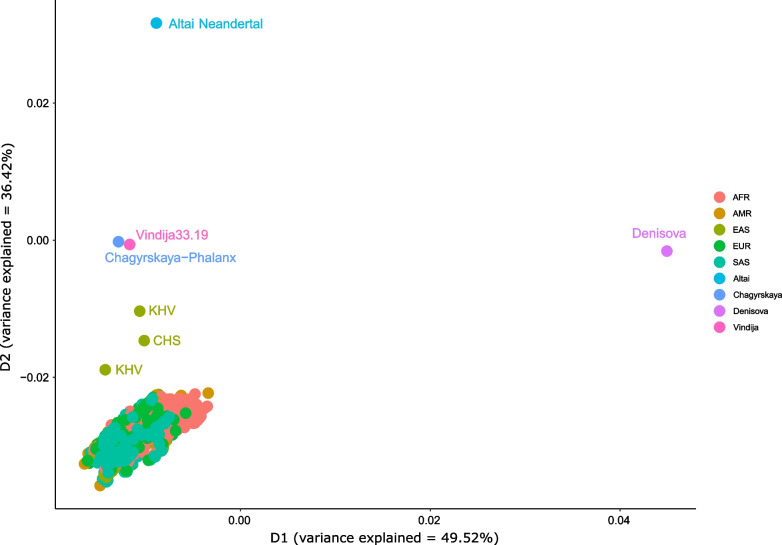
Weighted multidimensional scaling of archaic and EMH samples using the genetic variation present in *LRP5*. EMH samples have been weighted so all account for one fifth of the total weight. Each archaic sample accounts for one fifth of the total weight. AFR: African; AMR: Admixed American; EAS: East Asian; EUR: European; SAS: South Asian; KHV: Kinh in Ho Chi Minh City, Vietnam; CHS: Southern Han Chinese

Next, we analysed whether the presence of differential selective pressures in EMH could be explained by archaic introgression. First, we checked reported maps of archaic introgression in EMH. For the first map of introgression, generated from 27,566 Icelandic genomes [38], *LRP5* falls within a region of depletion of archaic introgression of 2.47 Mb, being one of the largest archaic-introgressed-free regions of the chromosome 11 (*p*-value = 0.0001). Analysis of a map of introgression of 1000G based on SPrime [37] supports the absence of signals of archaic introgression in populations out of Africa, with the exception of one CHS (Southern Han Chinese) and two KHV (Kinh in Ho Chi Minh City, Vietnam) haplotypes. In order to study this effect, we visualized the relationship between the introgressed haplotypes and the archaic populations. We constructed a genetic distance matrix between pairs of individuals using IBS. A weighted multidimensional scaling (wMDS) was run with this distance matrix by assigning the same weight to each of the four archaic samples, and dividing between all the 1000G samples the remaining weight (Fig. 2).

The first dimension (49.52% of explained variance) of the wMDS distinguishes the Denisovan sample against EMH and Neanderthal samples. The second dimension (36.42% of explained variance) distinguishes Altai Neanderthal against the rest. Interestingly, Vindija and Chagyrskaya cluster together and appear between EMH and Altai. Moreover, three haplotypes corresponding to CHS (Southern Han Chinese) and KHV (Kinh Vietnamese) populations appear as outliers from the EMH points, and closer to Vindija and Chagyrskaya.

### Evidences of different selective pressures in *LRP5* in EMH and archaic populations

**Fig. 3:**
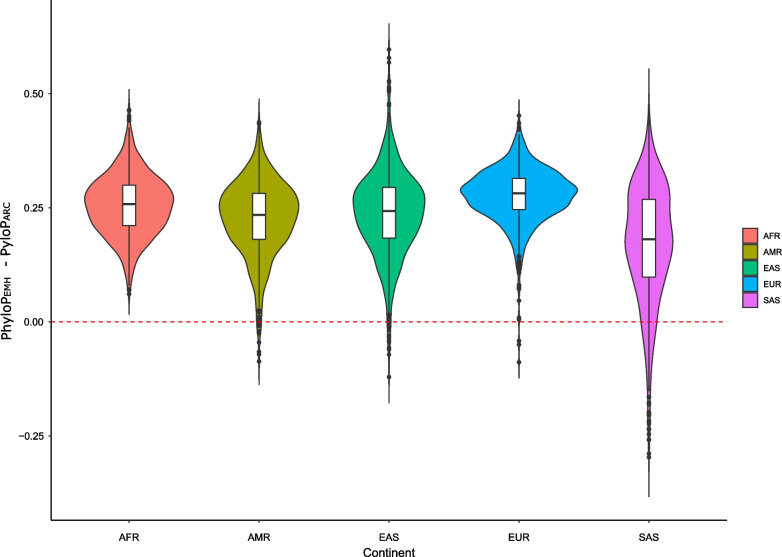
Violin plot of the distribution of the difference in the mean amount of PhyloP conservation at SNPs occurring in the EMH lineage of each continent compared to SNPs occurring in the ARC lineage. Each distribution for each continent was generated from 1000 datasets. Each dataset was obtained by sampling at random without replacement four individuals from the considered continent to match the number of archaic individuals, estimating the SNPs that occurred in the EMH lineage or in the ARC lineage, and computing the average PhyloP level of conservation. The red line indicates the expected value if SNPs occurred at each lineage on genomic positions with the same level of conservation. A value above 0 indicates that SNPs that occurred at the EMH lineage tend to happen in more conserved regions compared to ARC. AFR: African; AMR: Admixed American; EAS: East Asian; EUR: European; SAS: South Asian

Given the previous results, we wondered to which extent archaic populations and EMH populations showed evidence of different selective pressures. We used the map of nucleotide conservation among mammals (PhyloP) and the information of the ancestral allele of each SNP identified in EMH and archaic populations as defined in the 1000G to estimate the amount of conservation of SNPs that had appeared in the EMH genome compared to SNPs that appeared in the archaic populations. Our results (Fig. 3) show that SNPs that appeared (i.e., the derived allele is found) in the EMH lineage tend to occur in more conserved regions than SNPs from the archaic (ARC) lineage for all continental groups, except for South Asian (SAS) (*P*[mean conserved D_EMH > mean conserved D_ARC] in African (AFR) < 0.005, European (EUR) = 0.003, East Asian (EAS) = 0.014, Admixed American (AMR) = 0.014 and SAS = 0.102). Given that highly conserved regions tend to be associated with deleterious effects [60], these results would support the presence of different pressures acting on archaic populations compared to EMH populations. Specifically, purifying selection could exert a greater influence on ARC and/or a relaxation of selective pressures in EMH. Overall, all these results support a complex recent evolution of *LRP5*, with different selective pressures acting on archaic and EMH populations.

### Identification of putatively functional variants in *LRP5* in Neanderthals and Denisovans

**Fig. 4:**
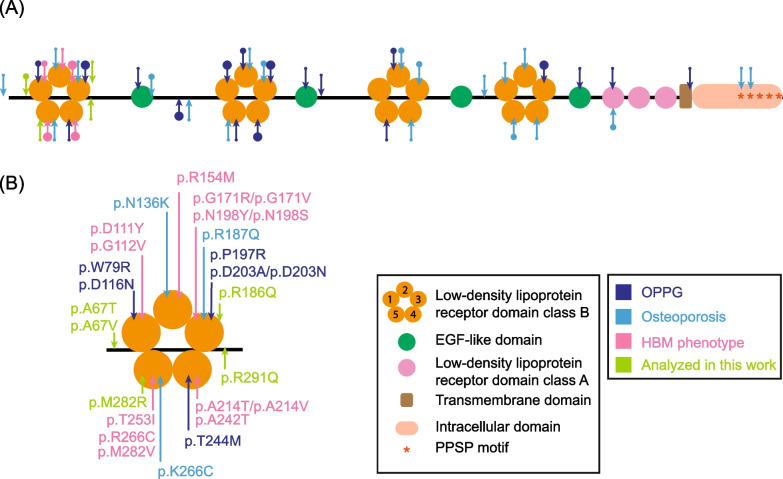
**A** Domain structure of the LRP5 co-receptor and localization of the missense variants associated with different human skeletal diseases (i.e., Osteoporosis pseudoglioma [OPPG], osteoporosis and HBM) according to the human gene mutation database (HGMD 2023.1), together with the archaic missense variants studied here. The arrows point to the location of the variants within the different LRP5 domains. The size of the points indicates the number of variants described in each domain: small points represent 1 variant, medium points represent 2 variants and large points represent 3 or more variants. **B** Zoom of the first β-propeller domain of the LRP5 protein (adapted from Martínez-Gil et al. 27)

Considering the previous results, we wondered whether ARC could harbor a particular set of *LRP5* variants compared to EMH populations. We searched available archaic *LRP5* genomic sequences and identified four missense variants in Neanderthals and one in the Denisovan individual (p.R291Q), all having a suggestive evidence of functionality (Table 1, Fig. 4): all of them are located in the first β-propeller; two of them (p.A67T and p.A67V) result in a change of the same protein residue but towards a different amino acid; a third one (p.R186Q) was found in two different Neanderthal individuals; and a fourth (p.M282R) affects a protein residue also mutated in EMH HBM cases.

**Table 1:** Archaic *LRP5* variants analyzed in this work

Genomic position (GRCh37)	Variant	Protein effect	gnomAD frequency	SIFT	Polyphen	Individual
chr11:68115422	G>A	p.A67T	7.08·10^–6^	0.0256	1.000	Vi33.26
chr11:68115423	C>T	p.A67V		0.001	1.000	Vi33.16
chr11:68125186	G>A	p.R186Q	4.60·10^–5^	0.000	1.000	Vi33.16, Vi33.25
chr11:68131373	T>G	p.M282R		0.039	0.999	Mez1
chr11:68131400	G>A	p.R291Q	1.22·10^–5^	0.012	0.995	Denisovan

### Structure-based functional analyses of the impact of LRP5 variants

**Fig. 5:**
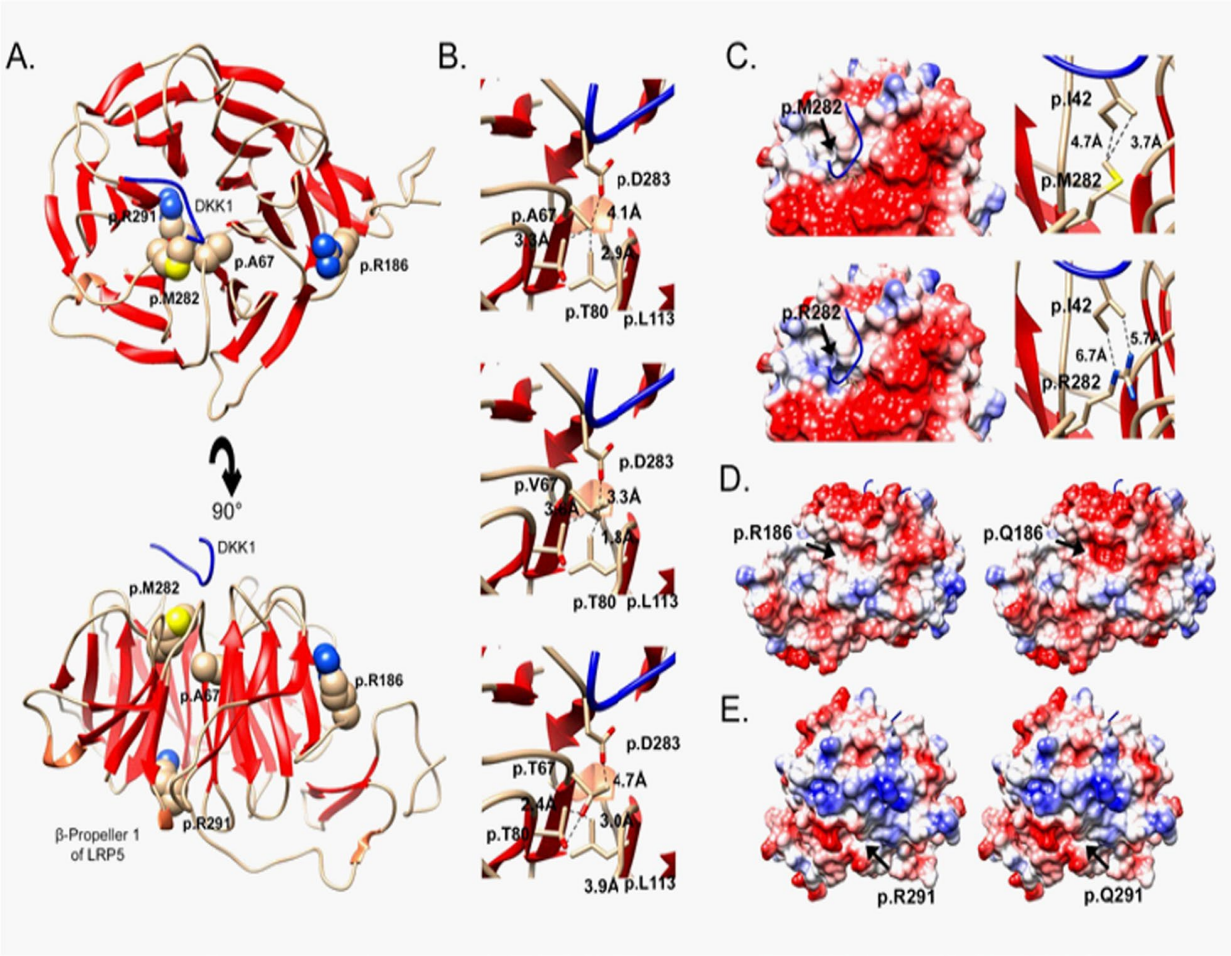
Molecular structure of LRP5. **A** Molecular homology model of the first β-propeller domain (at the top, top view, and at the bottom, side view). The residues mutated in Neanderthals and Denisovan are displayed in ball model and DKK1 is shown as a blue wire. **B** The substitution of Ala 67 by either Val or Thr affects the structure of the β-sheets due to steric hindrance. Residue 67 of LRP5 interacts with Asp 283 which is a key residue interacting with DKK1. **C** Detail of residue 282 of LRP5 interacting with isoleucine 42 of DKK1 (top: Met 282; bottom: Arg: 282; left: Solid electrostatic surface coloring; right: ribbon and wire display). **D** and **E** Evaluation of the substitutions Arg 186 by Gln and Arg 291 Gln by solid electrostatic surface coloring displays a decrease in the electrostatic charge in both cases (blue corresponds to positive charge and red to negative charge)

To determine the possible effect of the identified variants affecting residues p.A67, p.R186, p.M282 and p.R291, a protein homology model of the first β-propeller of LRP5 in interaction with DKK1 was generated (Fig. 5A). Interestingly, the p.A67 and p.M282 residues are located in the interaction region with DDK1 (Fig. 5A). For residue 67, we observe changes in the distances to p.D283, p.T80 and p.L113 in the mutated residues (Val or Thr), compared to the wild type (Ala), greater for Val than for Thr (Fig. 5B). In addition, the variants affect the structure of the β-sheets due to steric hindrance and cause changes in the stability of the protein [ΔΔG = 0.91 ± 0.02 kcal/mol (below the threshold of 1.6 kcal/mol, see methods) for the p.A67T and ΔΔG = 5.77 ± 0.10 kcal/mol for p.A67V]. In either case, no significant changes in interaction with DKK1 are observed (Supplementary Table S2).

The substitution of Met by Arg at position 282 causes three possible effects. On the one hand, a change in the surface electrostatic charge (Fig. 5C). Secondly, the atomic distances between the 282 residue of LRP5 and the I42 of DKK1 are longer with Arg than with Met, and the LRP5-DKK1 interaction ΔΔG is 2.7 kcal/mol (Fig. 5C, Supplementary Table S2). Finally, the protein stability ΔΔG is 6.16 ± 2.5 kcal/mol for p.M282R.

Variants p.R186Q and p.R291Q cause a change in the surface electrostatic charge (Fig. 5D, E) but do not affect protein stability (Supplementary Table S2).

Finally, we compared the protein stability changes (ΔΔG) of archaic variants with those of the HBM variants described in EMH and we did not observe any statistically significant difference between them (Supplementary Fig. S3).

### In vitro functional analysis of the impact of archaic LRP5 variants

**Fig. 6:**
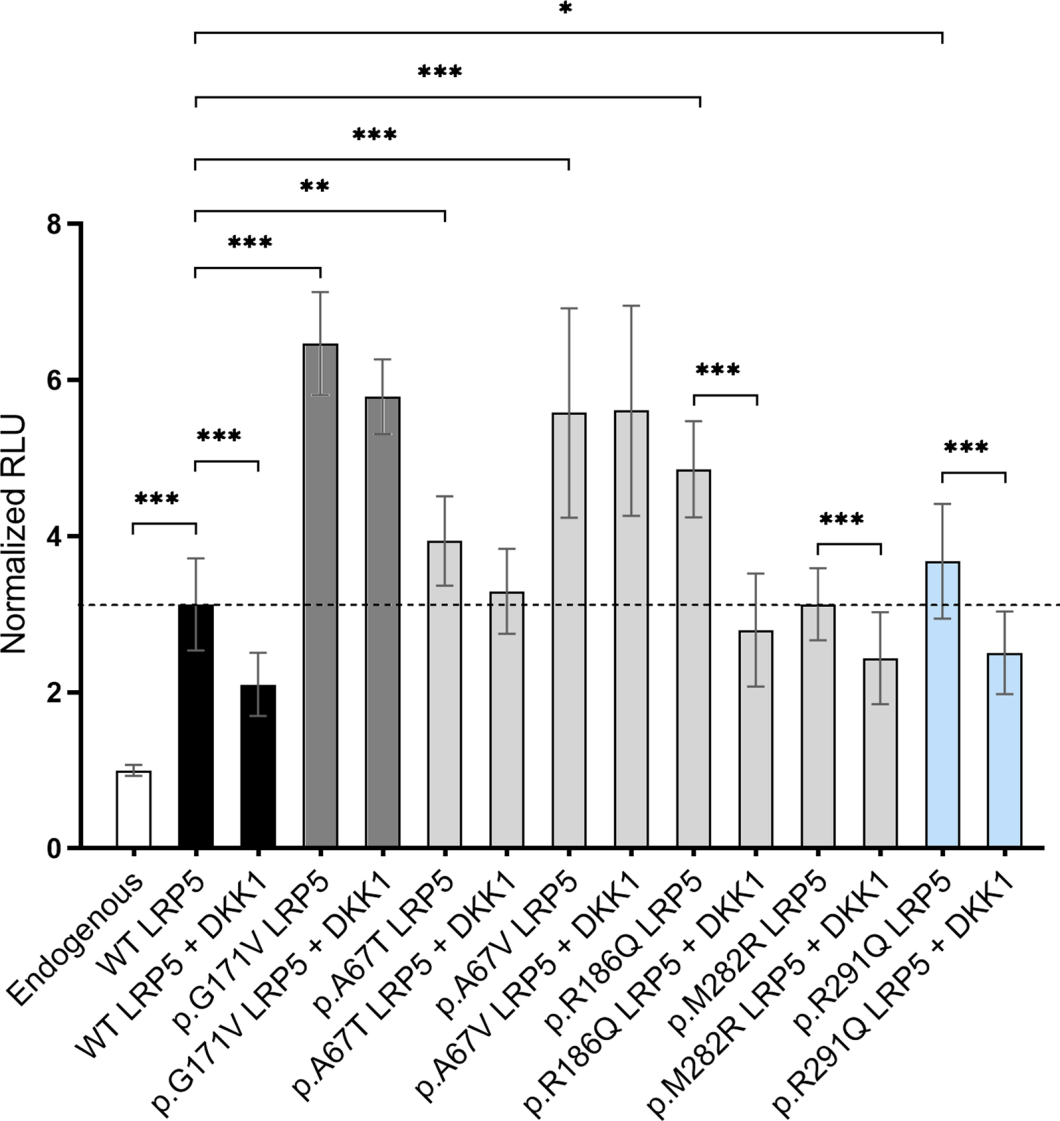
Relative luciferase activity of Wnt pathway for the endogenous pathway (empty vectors), the WT or LRP5-mutated active pathway (Wnt1, LRP5, mesd2), and the WT or LRP5-mutated inhibited pathway (Wnt1, LRP5, mesd2, DKK1), in Saos-2 cells. The white bar corresponds to the endogenous pathway, black bars correspond to WT LRP5, the dark grey bar correspond to the HBM-causing LRP5 mutation (used as positive control), light grey bars correspond to the LRP5 mutations identified in Neanderthals, blue bars correspond to the LRP5 mutation identified in Denisovan. Results are expressed as mean ± SD. *p < 0.05; **p < 0.01, ***p < 0.001

In order to evaluate the effect of the variants on the canonical Wnt pathway activity, we performed a luciferase reporter assay. Four of the variants (p.A67T, p.A67V, p.R186Q, and p.R291Q) displayed significantly greater Wnt pathway stimulation, compared to WT, with fold changes of 1.26 (*p*-value = 0.0013), 1.78 (*p*-value = 2.59·10^–10^), 1.55 (*p*-value = 2.59·10^–10^), and 1.18 (*p*-value = 0.0284), respectively, similar to the p.G171V variant (FC: 2.07; *p*-value = 2.59·10^–10^), used as positive control (Fig. 6). Moreover, for two of these variants (p.A67T and p.A67V) DKK1 failed to significantly inhibit Wnt pathway activation, similarly to p.G171V. No significant differences were observed between p.M282R and WT, either in the Wnt pathway activation or in the DKK1 inhibition.

## Discussion

It is well established that the *Homo* genus has undergone a skeletal gracilization, and particularly in EMH [1]. Such gracilization has been mainly explained by changes in the mechanical load in EMH [9]. However, GWAS studies using *loci* associated with BMD report differences between living human populations both in phenotype and genetics [16], raising new questions regarding which evolutionary processes, in terms of selective pressures and archaic introgression, have been at play. In the present study we focused on *LRP5*, one of the key genes regulating bone mass, which has been found mutated both in high and low bone mass phenotypes in EMH [23, 61]. We analyzed it from an evolutionary point of view and studied the structure and activity of some archaic variants.

The *LRP5* gene appears as one of the top genes in the popHumScan showing evidence of positive selection in populations from the 1000G. In particular, Sub-Saharan African and South Asian populations show evidence of positive selection events acting on different types of genetic variation. In the case of Sub-Saharan African populations, the popHumanScan database identifies an excess of adaptive variants, as exemplified by the α statistic, which could suggest the presence of ancient selective pressures. In the case of South Asian populations, recent signals of positive selection are derived from analyzing the patterns of linkage disequilibrium. All in all, this could imply the presence of multiple independent events of positive selection occurring in this gene, highlighting its evolutionary importance in EMH. Additional evidence of positive selection has been identified in the *LRP5* gene out of genes regulated by Vitamin D in East Asian populations from 1000G using frequency-spectrum-based tests [62]. Overall, these results suggest that the *LRP5* gene has a complex evolutionary history in human populations. When analyzing the role of archaic introgression, in our analyses only three haplotypes in East Asian populations from the 1000G are suggestive of archaic introgression. This result agrees with the map of introgression based on SPrime on the same samples [37]. Furthermore, Europeans from Iceland show an island of archaic introgression depletion at the *LRP5* gene region compared to the expectations under the null hypothesis of neutrality, thus supporting that hybridization has not been tolerated in this genomic region. Moreover, when analyzing the sites where mutations specific to each lineage occur, we observe that EMH populations tend to accumulate mutations at positions that are highly conserved in the primate lineage as defined by phyloP statistic [36], compared to variants present in the archaic lineage. Given that functional elements tend to be conserved across species [63], this result suggests that EMH and archaic populations have been under different selective pressures, with purifying selection exerting a greater influence on ARC and/or a relaxation of selective pressures in EMH, again highlighting that this *locus* seems to have undergone several independent events of selection. Moreover, if high BMD is the ancestral phenotype, then we would expect to identify high BMD variants in archaic populations after the split with Pleistocene modern humans. In silico and in vitro analyses can be devised on mutations specific to the archaic lineage to test this hypothesis. Considering that several heterozygous missense variants in the first β-propeller domain of LRP5 are described to cause HBM [27], we specifically looked for mutations in this region in archaic genomes and identified 5 potential mutations that met the selection criteria.

Human HBM mutations are gain-of-function changes that stimulate the Wnt pathway and reduce sclerostin and Dkk1 protein binding affinity and cell-based luciferase reporter systems have been extensively used to test them [59, 64–66]. Here, we took advantage of this system to find archaic mutations that similarly stimulate the Wnt pathway activity and gather in silico evidence supporting this, by creating a protein model. Two of the selected mutations (p.A67V and p.A67T) displayed the same in vitro effect as the well-known HBM p.G171V in agreement with in silico data, showing that they affect LRP5 stability. However, a loss of LRP5-DKK1 interaction was not observed in our structural model. Regarding p.R186Q and p.R291Q, our models displayed changes in surface electrostatic charges, which correlate with the luciferase results of higher pathway activation, but have no effect on the DKK1 inhibition. However, we did not observe any significant change in Wnt pathway activity and DKK1 inhibition for the p.M282R mutation even though the protein model predicted a triple effect changing the electrostatic charge, destabilizing the protein and the LRP5-DKK1 interaction. Interestingly, the comparison of the change in protein stability caused by archaic variants and modern human HBM mutations does not show any statistically significant difference, which might suggest that they have similar functional consequences.

Since we are modelling only small portions of LRP5 and DKK1, the discordance observed in some mutations between in vitro and in silico analyses may be explained by the fact that other LRP5 domains are involved in overall activity and DKK1 interaction, such as the third β-propeller [50, 67]. In addition, our static model might not fully represent the dynamic nature of LRP5 function. In this sense, the luciferase assay might better reflect the physiological context. On the other hand, the complexity of the assay used in this study, involving the cotransfection of several vectors, might have hindered differences in Wnt pathway activation below its sensitivity. Further studies in vivo would help to reinforce these results. We might envision a set of archaic LRP5 variants which would contribute to explain their robust skeletons, that did not introgress into EMHs.

Undoubtedly, focusing solely on the evolutionary, functional, and structural aspects of a single gene like *LRP5*, despite its pivotal role in BMD, cannot suffice to elucidate the complexity of HBM comprehensively. A similar approach should be extended to other genes implicated in the same phenotype. Additionally, our analysis has only encompassed genetic variants within the ARC identified in the (limited) available Neanderthal and Denisovan genomes. Considering the genetic diversity present in contemporary populations, it is plausible that the genetic variants examined within the ARC represent merely a fraction of the overall genetic variation in these populations, with much more yet to be uncovered. Moreover, as ancient genomic data from other hominins is being recovered, it can be expected that further analyses could be conducted to expand the conclusions of this study. Overall, this work opens the door to extend this kind of study to other genes involved in BMD determination.

## Conclusions

In conclusion, we provide data showing that *LRP5*, a gene with an important role in BMD determination, follows a complex evolutionary history both within EMH and between EMHs and archaic *Homo* species. This evolutionary history agrees with the complexity of the evolution of the skeletal phenotype. Our structural and in vitro analyses of archaic *LRP5* variants show that they resemble those causing HBM in EMH. Altogether, these data point to a genetic component that contributes to explain the skeletal differences between EMH and archaic human populations.

### Supplementary Information

Additional file1 

## Data Availability

The protein model underlying this article is available in Model Archive (10.5452/ma-1smp3).
