# Supplementary material for: Evolutionary and functional analyses of LRP5 in archaic and extant modern humans
Source: Hum Genomics. 2024 May 27;18:53. doi: 10.1186/s40246-024-00616-6 (PMC11131306; doi:10.1186/s40246-024-00616-6)
Supplement: Supplementary file 1 — Additional file1 [file 40246_2024_616_MOESM1_ESM.docx]

**Supplementary Information**


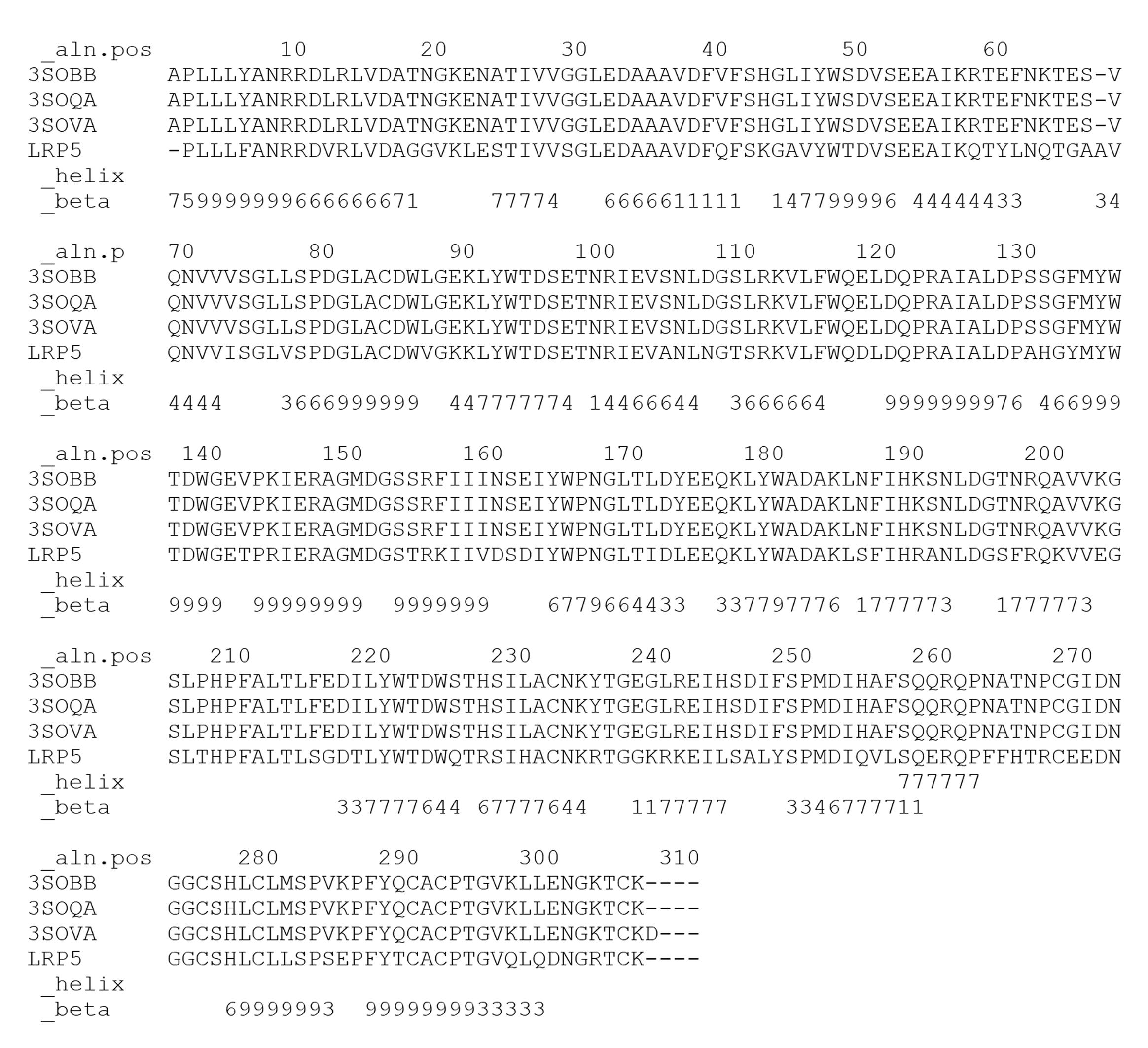


**Supplementary Fig. S1. Alignment of sequence template and LRP5 sequence to generate de MHM**

**A**
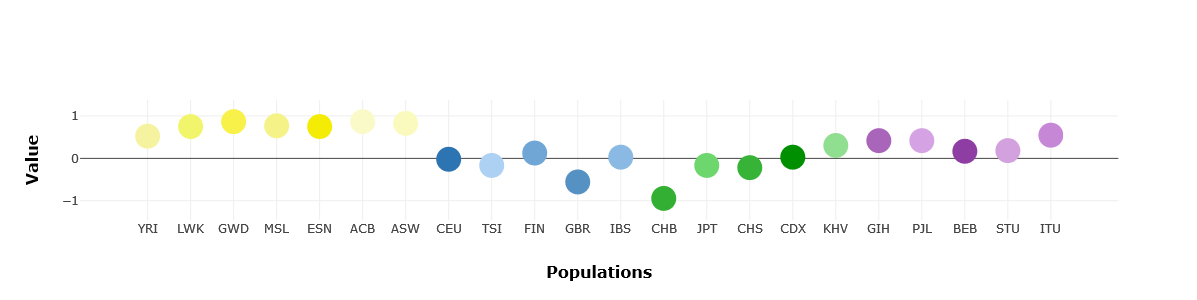


**B**


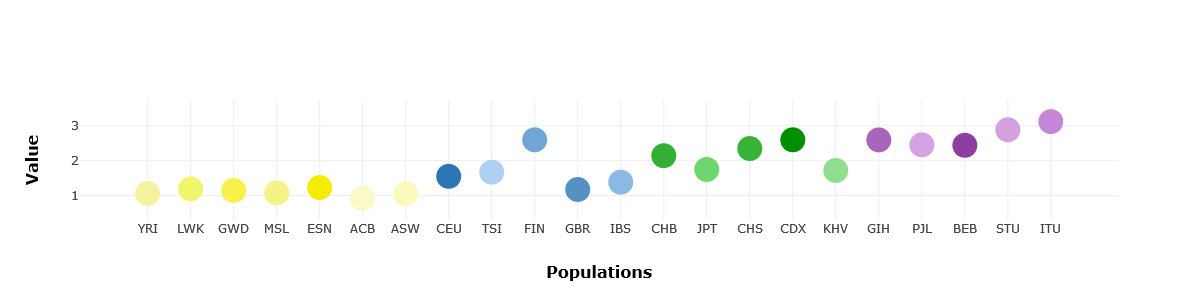


**Supplementary Fig. S2**. Alpha (A) and iHS (B) statistics in the *LRP5* gene for the 1000G populations computed by popHumanScan. Sub-Saharan African populations (yellow) show large values for the alpha statistic. South Asian populations (purple) show large iHS values. In both cases, these values are compatible with the fingerprint of positive selection. YRI: Yoruba in Ibadan, Nigeria; LWK: Luhya in Webuye, Kenya; GWD: Gambian in Western Division; MSL: Mende in Sierra Leone; ESN: Esan in Nigeria; ACB: African Caribbean in Barbados; ASW: American's of African Ancestry in SW USA; CEU: Utah Residents (CEPH) with Northern and Western European ancestry; TSI: Toscani in Italia; FIN: Finnish in Finland; GBR: British in England and Scotland; IBS: Iberian population in Spain; CHB: Han Chinese in Beijing; JPT: Japanese in Tokyo; CHS: Southern Han Chinese; CDX: Chinese Dai in Xishuangbanna; KHV: Kinh in Ho Chi Minh City, Vietnam; GIH: Gujarati Indian from Houston, Texas; PJL: Punjabi from Lahore, Pakistan; BEB: Bengali from Bangladesh; STU: Sri Lankan Tamil from the UK; ITU: Indian Telugu from the UK


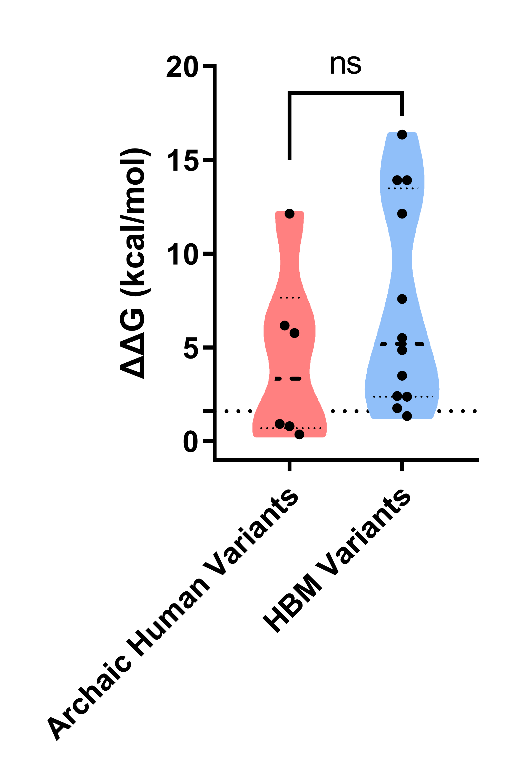


**Supplementary Fig. S3.** Stability ΔΔG±SD (kcal/mol) of variants in Archaic humans and High Bone Mass (HBM) variants in the LRP5 protein. The dot line indicate the threshold established at 1.6 kcal/mol. Dash lines indicate median and quartiles.

**Supplementary Table S1. X-Ray crystallography structures evaluated from PDB**

|  | 3S94 | 3SOB | 3SOQ | 3SOV | 4DG6 | 5GJE |
| --- | --- | --- | --- | --- | --- | --- |
| Method | X-ray  crystallography | X-ray  crystallography | X-ray  crystallography | X-ray  crystallography | X-ray  crystallography | Electron Microscopy |
| Resolution [Å] | 2.8 Å | 1.9 Å | 1.9 Å | 1.27 Å | 2.9 Å | 21.0 Å |
| R-Value  (Free // Work) | 0.296 // 0.237 | 0.212 // 0.174 | 0.221 // 0.177 | 0.180 // 0.153 | 0.313 // 0.257 | - |
| Chains | A, B | B | A | A | A | A |
| Length | 305 | 305 | 305 | 305 | 305 | 305 |
| E-value | 5.84961E-132 | 1.68734E-132 | 1.59161E-132 | 1.59161E-132 | 3.91915E-132 | 5.84961E-132 |
| % Identity of B-propeller 1 & EGF-L1 | 221/305 (72%) | 221/305 (72%) | 221/305 (72%) | 221/305 (72%) | 221/305 (72%) | 221/305 (72%) |
| Score | 468.774bits (1205) | 470.7bits (1210) | 470.7bits (1210) | 470.7bits (1210) | 469.159bits (1206) | 468.774bits (1205) |
| Positives | 259/305 (85%) | 259/305 (85%) | 259/305 (85%) | 259/305 (85%) | 259/305 (85%) | 259/305 (85%) |
| Gaps | 1/305 (0%) | 1/305 (0%) | 1/305 (0%) | 1/305 (0%) | 1/305 (0%) | 1/305 (0%) |
| Structure  region | B-Propeller 1 y 2  EGF-like 1 y 2 | B-Propeller 1  EGF-like 1 | B-Propeller 1  EGF-like 1 | B-Propeller 1  EGF-like 1 | B-Propeller 1 y 2  EGF-like 1 y 2 | B-Propeller 1,2,3 y 4  EGF-like 1,2,3 y 4 |
| Protein | LRP6 | LRP6 | LRP6 | LRP6 | LRP6 | LRP6 |
| PMID | [**21984209**](http://www.rcsb.org/pdb/search/smartSubquery.do?smartSearchSubtype=PubmedIdQuery&pubMedIdList=21984209) | [**21944579**](http://www.rcsb.org/pdb/search/smartSubquery.do?smartSearchSubtype=PubmedIdQuery&pubMedIdList=21944579) | [**21944579**](http://www.rcsb.org/pdb/search/smartSubquery.do?smartSearchSubtype=PubmedIdQuery&pubMedIdList=21944579) | [**21944579**](http://www.rcsb.org/pdb/search/smartSubquery.do?smartSearchSubtype=PubmedIdQuery&pubMedIdList=21944579) | [**22696217**](https://www.ncbi.nlm.nih.gov/pubmed/22696217) | [**28052259**](http://www.rcsb.org/pdb/search/smartSubquery.do?smartSearchSubtype=PubmedIdQuery&pubMedIdList=28052259) |

**Supplementary Table S2. Effect of LRP5 archaic and HBM variants**

| **Variants** | **Repeat*** | **Effect** | **ΔΔG Stability ± SD LRP5 (kcal/mol)^1^** | **ΔΔG Interaction LRP5-DKK1 (kcal/mol)** |
| --- | --- | --- | --- | --- |
| Archaic Human Variants | | | | |
| p.A67T |  | Stability? | 0.91 ± 0.02 | -0.05 |
| p.A67V |  | Stability | **5.77 ± 0.10** | -0,09 |
| p.R186Q | LDL-receptor class B 3 | Electrostatic | 0.80 ± 0.1 | NA^2^ |
| p.M282R | LDL-receptor class B 5 | Stability/Interaction/  Electrostatic | **6.16 ± 2.5** | 2.7 |
| p.R291Q |  | Electrostatic | 0.36 ± 0.47 | NA |
| HBM Variants | | | | |
| p.D111Y | LDL-receptor class B 1 | Stability | **-1.76 ± 0.60** | NA |
| p.G112V | LDL-receptor class B 1 | Stability | **7.59 ± 0.01** | NA |
| p.R154M | LDL-receptor class B 2 | Interaction | 0.93 ± 0.18 | ? |
| p.G171R | LDL-receptor class B 3 | Stability | **13.92 ± 2.60** | NA |
| p.G171V^#^ | LDL-receptor class B 3 | Stability | **12.14 ± 0.43** | NA |
| p.N198Y | LDL-receptor class B 4 | Stability | **16.35 ± 1.67** | NA |
| p.N198S | LDL-receptor class B 4 | Stability | 1.34 ± 0.43 | NA |
| p.A214T | LDL-receptor class B 4 | Stability | **4.86 ± 0.21** | NA |
| p.A214V | LDL-receptor class B 4 | Stability | **5.50 ± 0.17** | NA |
| p.A242T | LDL-receptor class B 5 | Stability | **2.37 ± 0.49** | NA |
| p.T253I | YWTD4 | Stability | **3.48 ± 0.85** | NA |
| p.R266C | LDL-receptor class B 5 | Electrostatic | 0.68 ± 0.18 | NA |
| p.M282V | LDL-receptor class B 5 | Stability | **2.40 ± 0.52** | NA |

*All variants are found in the first β-propeller region.

^#^Variant used as positive control for the luciferase assay.

**^1^**Threshold: ±1.6 kcal/mol (corresponding to twice the standard deviation calculated with FoldX). Values above threshold are indicated in bold).

**^2^**The residue is not located at the DKK1 interaction domain of LRP5 in our model.
